# Knowledge and attitude towards pregnancy-related issues of Zika virus infection among general practitioners in Indonesia

**DOI:** 10.1186/s12879-019-4297-4

**Published:** 2019-08-06

**Authors:** Harapan Harapan, Yogambigai Rajamoorthy, Prattama S. Utomo, Samsul Anwar, Abdul M. Setiawan, Alma Alleta, Alfredo Bambang, Muhammad R. Ramadana, Ikram Ikram, Nur Wahyuniati, Reza Maulana, Ichsan Ichsan, Rosaria Indah, Abram L. Wagner, Ulrich Kuch, David A. Groneberg, Alfonso J. Rodríguez-Morales, Mohd Andalas, Ruth Müller, Mudatsir Mudatsir, Allison Imrie

**Affiliations:** 1 Medical Research Unit, School of Medicine, Universitas Syiah Kuala, Banda Aceh, Indonesia; 2 Tropical Disease Centre, School of Medicine, Universitas Syiah Kuala, Banda Aceh, Indonesia; 3 Department of Microbiology, School of Medicine, Universitas Syiah Kuala, Banda Aceh, Indonesia; 4 School of Biomedical Sciences, The University of Western Australia, Nedlands, Australia; 5 Universiti Tunku Abdul Rahman, Kajang, Selangor Malaysia; 6 Department of Medical Education, Faculty of Medicine, Gadjah Mada University, Jogjakarta, Indonesia; 7 Department of Statistics, Faculty of Mathematics and Natural Sciences, Universitas Syiah Kuala, Banda Aceh, Indonesia; 8 Department of Microbiology, Faculty of Medicine and Health Sciences, Maulana Malik Ibrahim State Islamic University of Malang, Malang, Indonesia; 9 Department of Family Medicine, School of Medicine, Universitas Syiah Kuala, Banda Aceh, Indonesia; 10 Department of Microbiology, School of Medicine, Atma Jaya Catholic University of Indonesia, Jakarta, Indonesia; 11 Medical Education Unit, School of Medicine, Universitas Syiah Kuala, Banda Aceh, Indonesia; 12 Sydney School of Education and Social Work, Faculty of Arts and Social Sciences, The University of Sydney, Sydney, Australia; 13 Department of Epidemiology, University of Michigan, Ann Arbor, MI USA; 14 Institute of Occupational Medicine, Social Medicine and Environmental Medicine, Goethe University, Frankfurt am Main, Germany; 15 Public Health and Infection Research Incubator and Group, Faculty of Health Sciences, Universidad Tecnológica de Pereira, Pereira, Risaralda Colombia; 16 Research Group Medical and Diagnostic Images (GRIMEID), IPS Imágenes Diagnósticas S.A, Pereira, Risaralda Colombia; 17 Department of Obstetrics and Gynecology, School of Medicine, Universitas Syiah Kuala, Banda Aceh, Indonesia; 18 Unit of Medical Entomology, Institute of Tropical Medicine, Antwerp, Belgium

**Keywords:** Zika virus, Knowledge, Attitude, General practitioner, Healthcare worker

## Abstract

**Background:**

The aim of this study was to assess the knowledge and attitudes towards pregnancy-related issues of Zika virus (ZIKV) infection among general practitioners (GPs), a frontline healthcare worker group, in Indonesia.

**Methods:**

A cross-sectional, online survey assessing knowledge and attitudes towards ZIKV infection on multiple-item scales was sent to GPs in the Sumatra and Java islands of Indonesia. The associations between independent factors and either knowledge or attitude were assessed with logistic regressions. The correlation and association between knowledge and attitude were estimated.

**Results:**

We included 457 (53.7%) out of 850 responses in the analysis. Among these, 304 (66.5%) and 111 (24.2%) respondents had a good knowledge and attitude, respectively. No demographic, workplace, professional development, or experiential characteristics related to ZIKV infection were associated with knowledge. In the multivariate analysis, only contact experience was associated with attitude. There was a significant, positive correlation between knowledge and attitude scores.

**Conclusions:**

Although knowledge of pregnancy-related complications of ZIKV infection is relatively high among GPs in Indonesia, more than 75% of them had a poor attitude towards pregnancy-related issues of Zika. Strategies for enhancing the capacity of GPs to develop positive attitudes and respond to ZIKV infection are needed.

**Electronic supplementary material:**

The online version of this article (10.1186/s12879-019-4297-4) contains supplementary material, which is available to authorized users.

## Background

Zika virus (ZIKV) is transmitted by *Aedes aegypti* and *Aedes albopictus* mosquitoes. Infection by ZIKV most often results in mild symptoms such as fever, maculopapular rash, arthralgia, cephalgia and conjunctivitis [1]. However, the infection may cause severe complications including neurological sequelae and Guillain-Barré syndrome [2]. In addition, ZIKV-related congenital microcephaly, ventriculomegaly and intracranial calcification have been identified in infants born to ZIKV-positive mothers [3]. There is a strong association between congenital ZIKV infection and microcephaly; one study estimates the odds of microcephaly to be 73.1 times higher among those with ZIKV infection [4]. Multiple outbreaks of microcephaly-associated with Zika cases have been reported since reemerge [5–9]. In Brazil for example, during two Zika outbreaks in 2015 and 2016, more than 1.6 million cases were reported and during this period, 1950 cases of ZIKV infection-related microcephaly were confirmed [9]. This devastating complication was one factor that led the WHO to declare ZIKV infection as a Public Health Emergency of International Concern (PHEIC) and as an ongoing challenge in 2016 [10]. Microcephaly associated with ZIKV infection has been also reported in Asian countries [11–13].

ZIKV was first found in Africa over 70 years ago when it was identified in a monkey in 1947 [14] and in humans in 1954 [15]. After local outbreaks in various Pacific Islands [16], the virus reached the Americas (Brazil) in 2015 [17], and has since continued to spread and has been reported in 86 countries in the Americas, Africa, and Southeast Asia [18]. Globally, it is predicted that over 2.17 billion people live in areas that are environmentally suitable for ZIKV transmission, and 1.42 billion of them live in Asia [19]. Asia is susceptible to epidemic ZIKV transmission because of widespread distribution of the mosquito vectors for ZIKV, the large amount of travel to and from Zika-affected areas, conducive conditions for ZIKV transmission, and limited health resources [20–23].

A recent modeling study ranked Indonesia as the third country most at risk for ZIKV exposure due to the monthly volume of airline travelers [21]. Indonesia has not yet had a Zika outbreak, but there is evidence of ZIKV transmission in the country. Studies using serum samples from 1978 and 1983 found evidence of anti-ZIKV antibodies in Central Java and Lombok [24, 25]. In 2014, 9.1% of serum samples from children 1–4 years old were ZIKV seropositive [26]. In 2013 and 2014, two travelers visiting Indonesia acquired ZIKV infection and were diagnosed after returning to Australia [27, 28]. In 2015, an individual with no history of traveling abroad was diagnosed with Zika [29]. Zika cases may be underestimated in Indonesia for three major reasons: (1) the clinical presentation of Zika and dengue is similar; (2) the availability of Zika diagnostic tests is limited in Indonesia; and (3) Zika reports are based on passive case-based surveillance [30, 31].

In order to mitigate Zika complications, in particular pregnancy-related complications, it is important to diagnose the disease in its early stages and implement prevention programmes. Assessment of the knowledge about ZIKV infection among healthcare workers (HCWs) can identify problems that HCWs may face in reaching this diagnosis. Zika-related knowledge is important since knowledge gaps may lead to attitudes and practices that hamper ZIKV infection diagnosis, management, and risk reduction [32].

Studies related to knowledge and attitude towards ZIKV infection have been conducted in various community groups. At least 21 studies related to knowledge of ZIKV infection have been conducted [1, 33–52], in the general population [33–37] or specific groups such as reproductive-age women [38, 39], pregnant women [40–43], construction workers [44], and medical [1, 39, 45–49, 52] and non-medical university students [33, 52]. Five of these studies were conducted among HCWs [1, 39, 45, 50, 51]. Ten studies related to attitude towards ZIKV infection have been conducted [34, 35, 37, 41, 48, 52–55], but only one of these among HCWs [56]. These figures indicate that information about knowledge and attitude towards ZIKV infection among HCWs is limited. Given the fact that HCWs have critical roles in the prevention and management of Zika disease, studies to fill this gap in the literature are required.

In Indonesia, general practitioners (GPs) are the frontline HCWs who are responsible for the early diagnosis, treatment, and prevention of diseases including ZIKV infection. Thus, GPs in Indonesia should have some knowledge to treat ZIKV infection because it has the general manifestation of fever, which is included in the competency list of general medical practice [57]. In addition, the symptoms of ZIKV infection are similar to those of other mosquito-borne infections such as dengue fever or chikungunya fever [58]. It is important for GPs to recognize symptoms of ZIKV infection in order to detect or prevent complications. Most Zika prevention measures, including the strategic responses set out by the WHO, are also within the GPs’ competency list. These circumstances indicate that GPs play a pivotal role in the early detection and prevention of ZIKV infection in Indonesia.

Previously, our group conducted a project to assess the knowledge and attitude towards ZIKV infection among doctors in Aceh province of Indonesia [51, 56]. Both specialist doctors and GPs were included, and multiple stratified analyses indicated that specialists had a lower knowledge and a less positive attitude compared to GPs. To generalize these results in the national context, an expanded survey that included other regions of Indonesia was conducted. As GPs are the frontline HCWs in Indonesia, and because one of the most important issues related to ZIKV infection is congenital microcephaly, the survey sampled GPs and focused on the pregnancy-related issues of ZIKV infection. Thus, this study sought to provide a comprehensive picture of the knowledge and attitude towards pregnancy-related issues of ZIKV infection among GPs in Indonesia.

## Methods

### Study design and survey instrument

Following our previous survey of HCWs in Aceh province [51, 56], another survey, from October to December 2016, on GPs’ knowledge and attitude towards pregnancy-related issues of ZIKV infection was conducted in a larger area (encompassing Sumatra and Java islands, which together are inhabited by about 200 million individuals, or approximately 80% of the entire population of Indonesia). This study was cross-sectional and used an online survey which was estimated to take around five to ten minutes to complete. To assess knowledge and attitude, a questionnaire was developed based on existing facts from the United States Centers for Disease Control and Prevention (CDC) [17]. The questions were translated into Bahasa Indonesia (national language), and a panel consisting of two medical microbiologists was appointed to evaluate the validity of the questionnaire content. Prior to the actual study, the reliability of the questions was tested in a pilot study, and the data from this pilot study were excluded from the final analysis. The Cronbach’s alpha score was 0.78 and 0.70 for knowledge and attitude domain, respectively, indicating good internal consistency of the items in the scale. The design, setting, analyses and reporting of this study adhered to the STROBE guidelines for cross-sectional studies in epidemiology (see Additional file 1 for the detailed checklist of STROBE criteria [59]).

### The survey and data collection

The Survey Monkey web portal was employed to collect the data for this study. A survey link was sent to physicians’ professional organizations that forwarded it to their members and promoted the survey on social media. All registered GPs from Indonesian Medical Council living in Sumatera or Java were considerate eligible. If no response was received from a member, reminders were sent. To ensure the anonymity of the respondent and confidentiality of the data, the IP addresses of participants were not collected, and only the principal investigator had access to the survey account. The first page of the survey included information on the identity of the principal investigator, contact details, collaborating institutions, aims of the study, and its expected benefits. Before starting the survey, individuals provided their consent to participate. Potential participants were also informed that they could exit the survey at any point, but their responses that had already been recorded would still be uploaded to the database. At the end of the data collection period, the data were extracted from the survey host and imported into statistical software for analysis.

### Measures

#### Dependent variables

The dependent variables in this study were knowledge and attitude among GPs in Indonesia towards pregnancy-related problems of ZIKV infection. To assess knowledge, five questions were used. A correct response was given a score of one and an incorrect response was given a score of zero. The knowledge score was the sum of these five questions, ranging from 0 to 5 in which higher scores indicated greater knowledge. Four questions were used to assess attitude, and each question was rated on a 5-point Likert scale from 1 (strongly disagree) to 5 (strongly agree). To describe the distribution of responses to a question in the study population, participants who answered 4 or 5 were categorized as “agreeing” with the specific question. Subsequently, responses within the attitude domain using the 1 to 5 scale were added together giving an attitude score ranging from 4 to 20 in which higher scores indicated a more positive attitude. The responses to questions in which a higher score indicated a more negative attitude were flipped in order to preserve directionality across questions. See Additional file 2 for the detailed questions used to assess the knowledge and attitude domain.

#### Independent variables

We assessed four main groups of independent variables that could plausibly affect knowledge and attitude: sociodemographic status, workplace characteristics, medical professional development characteristics, and Zika-related experience. The sociodemographic characteristics included gender, age, education, and occupation. For statistical analysis purposes, the age of the respondents was divided into (a) 30 years or less, and (b) more than 30 years. Workplace characteristics included the specific department where the respondents worked, workplace type, and workplace location. To assess their medical professional development characteristics, respondents were asked about their medical experience (in year) and experience attending medical conference or training in the last five months. Zika-related experience was assessed whether respondents had contact with patient(s) presenting signs and symptoms of ZIKV infection.

### Statistical analysis

All explanatory variables were divided into groups to give quantitative measures. The levels of knowledge and attitude were dichotomized into “good” and “poor” based on a 75% cut-off point of the total score within each domain (i.e., 4 out of 5, and 15 out of 20 for knowledge and attitude, respectively). Our studies have used a similar cut-off point of either 75% or 80% [51, 56, 60–65]. The associations between the independent variables and the dependent variables (knowledge and attitude) were assessed using a two-step logistic regression. In the univariate logistic regression, all explanatory variables were analyzed, and variables with *P* ≤ 0.25 in this step were entered into the multivariate analysis. The estimated crude odds ratio (OR) of the univariate analyses and the adjusted OR (aOR) of the multivariate analyses were interpreted in relation to a reference category (R).

The correlation and association between knowledge and attitude were assessed using Spearman’s rank correlation (r_s_) and the Chi-square test, respectively. This correlation analysis was used due to the homogeneity of data variance. The confidence intervals for Spearman’s rank correlation were calculated as previously described [66]. For all analyses, significance was assessed at α = 0.05. Statistical Package of Social Sciences 17.0 software (SPSS Inc., Chicago, IL, USA) was used to analyze the data.

## Results

### Respondents’ characteristics

We received 850 responses during the survey period. Among these, 393 responses had to be excluded due to incomplete information, leaving a total of 457 (53.7%) participants with complete responses. Approximately 60% of the participants were 30 years old or younger, and 66.3% of them had a medical practice experience of less than five years (Table 1). Approximately 10% of the respondents had an additional higher degree such as a master’s or doctoral degree. The number of women was 16% higher than the number of men (58.0% vs. 42.0%). The occupation of 12.3% of the respondents included an appointment both as a GP and university staff, and less than 10% of them were specialist resident. A third of the participants were working in emergency units, and approximately the same number was working in community health centres (*Puskesmas*). The majority (70.3%) of the GPs enrolled in this survey were working in a health centre located in the rural (district) or sub-urban (regency) areas, and about 30% of them were working in the urban areas (provincial level). Although 66.1% of the participants had attended a provincial conference, 93.0% of them had not attended an international conference within the last five months. Approximately 8% of the surveyed GPs had had contact with a patient presenting signs and symptoms of ZIKV infection.

**Table 1:** Univariate and multivariate logistic regression analysis showing predictors of knowledge about Zika virus infection (good vs. poor) (*N* = 457)

Variable	*n* (%)	Good knowledge *n* (%)	Univariate		Multivariate	
			OR (95% CI)	*P*–value	aOR (95% CI)	*P*–value
Location						
Aceh (R)	365 (79.9)	245 (67.1)	1			
Non-Aceh	92 (20.1)	59 (64.1)	0.88 (0.54–1.41)	0.587		
Gender						
Male (R)	192 (42.0)	128 (66.7)	1			
Female	265 (58.0)	176 (66.4)	0.99 (0.67–1.47)	0.995		
Age group						
30 years or less (R)	279 (61.1)	183 (65.6)	1			
More than 30 years	178 (38.9)	121 (68.0)	1.11 (0.75–1.66)	0.598		
Education						
General practitioner (R)	407 (89.1)	267 (65.6)	1		1	
GP with master’s or doctoral degree	50 (10.9)	37 (74.0)	1.49 (0.77–2.90)	0.237	1.20 (0.55–2.63)	0.642
Occupation						
GP and non-university staff (R)	356 (77.9)	235 (66.0)	1		1	
GP and university staff	56 (12.3)	43 (76.8)	1.70 (0.88–3.29)	0.113	1.47 (0.67–3.21)	0.334
Specialist residency	45 (9.8)	26 (57.8)	0.71 (0.38–1.32)	0.227	0.61 (0.30–1.22)	0.162
Department						
Community health centre (R)	162 (35.4)	103 (63.6)	1			
Emergency unit	136 (29.8)	95 (69.9)	1.33 (0.82–2.16)	0.254		
Others	159 (34.8)	106 (66.7)	1.15 (0.72–1.81)	0.562		
Type of workplace						
Community health centre (R)	95 (20.8)	57 (60.0)	1		1	
Private clinic or hospital	191 (41.8)	133 (69.6)	1.53 (0.92–2.56)	0.105	1.39 (0.82–2.36)	0.222
Government hospital	171 (37.4)	114 (66.7)	1.33 (0.79–2.24)	0.277	1.50 (0.86–2.60)	0.153
Location of workplace						
District (R)	137 (30.0)	87 (63.5)	1			
Regency	184 (40.3)	123 (66.8)	1.16 (0.73–1.84)	0.533		
Province	136 (29.7)	94 (69.1)	1.29 (0.78–2.13)	0.327		
Attended province-level conference						
Yes	302 (66.1)	202 (66.9)	1.05 (0.70–1.58)	0.817		
No (R)	155 (33.9)	102 (65.8)	1			
Attended national conference						
Yes	160 (35.0)	104 (65.0)	0.90 (0.60–1.35)	0.613		
No (R)	297 (65.0)	200 (67.3)	1			
Attended international conference						
Yes	32 (7.0)	20 (62.5)	0.83 (0.39–1.74)	0.618		
No (R)	425 (93.0)	284 (66.8)	1			
Medical practice experience (years)						
Less than 5 years (R)	303 (66.3)	197 (65.0)	1			
5 years or more	154 (33.7)	107 (69.5)	1.23 (0.81–1.86)	0.340		
Had contact with patient(s) presenting signs and symptoms of Zika virus infection						
Yes	36 (7.9)	22 (61.1)	0.78 (0.39–1.56)	0.475		
No (R)	421 (92.1)	282 (67.0)	1			

*aOR* Adjusted odds ratio, *CI* Confidence interval, *OR* Odds ratio, *R* Reference group

### Knowledge and associated determinants

We rated 304 (66.5%) respondents as having a good knowledge about pregnancy-related issues with ZIKV infection. Approximately 88.5% knew that ZIKV can be passed from a pregnant woman to the fetus, and 92.7% knew that a baby could have congenital defects if infected during pregnancy. Interestingly, although 416 (91.0%) correctly mentioned that microcephaly was a congenital condition associated with ZIKV infection, 65.8% of the respondents also answered that a ventricular septal defect was a complication associated with ZIKV infection. We assessed the association between knowledge and independent factors from four domains (sociodemographic status, workplace characteristics, professional development characteristics, and experience related to Zika disease). No factor was associated with knowledge (Table 1).

### Attitude and associated determinants

Although the knowledge level of the respondents was relatively high, only 111 (24.2%) of them had a positive attitude. Sixty percent of the respondents believed that women with ZIKV infection should undergo Caesarean section, or were not sure whether they should. Although more than 65% of the respondents believed that antibiotic treatment was not required for women with ZIKV infection, 107 (23.4%) of them were still confused about this treatment option. In addition, almost 50% of the respondents believed that pregnant women with ZIKV infection should be treated with an antiviral drug, and 116 (25.3%) were unsure whether antiviral medication was required.

Among the four domains of independent factors that were assessed, older age and contact experience with a patient presenting signs and symptoms of ZIKV infection were associated with a positive attitude in the univariate analysis (OR: 1.87, 95% CI: 1.21–2.88, *P* = 0.005, and OR: 3.53, 95% CI: 1.76–7.05, *P* < 0.001, respectively). The multivariate analysis revealed that only contact experience was significantly associated with attitude (OR: 3.14, 95% CI: 1.49–6.58, *P* = 0.003) (Table 2).

**Table 2:** Univariate and multivariate logistic regression analysis showing predictors of attitude towards Zika virus infection (good vs. poor) (*N* = 457)

Variable	*n* (%)	Good attitudes *n* (%)	Univariate		Multivariate	
			OR (95% CI)	*P*–value	aOR (95% CI)	*P*–value
Location						
Aceh (R)	365 (79.9)	84 (23.0)	1		1	
Non-Aceh	92 (20.1)	27 (29.3)	1.39 (0.83–2.32)	0.207	1.31 (0.75–2.27)	0.341
Gender						
Male (R)	192 (42.0)	54 (28.1)	1		1	
Female	265 (58.0)	57 (21.5)	0.70 (0.46–1.08)	0.104	0.64 (0.40–1.03)	0.065
Age group						
30 years or less (R)	279 (61.1)	55 (19.7)	1		1	
More than 30 years	178 (38.9)	56 (31.5)	1.87 (1.21–2.88)	0.005*	1.59 (0.78–3.21)	0.200
Education						
General practitioner (GP) (R)	407 (89.1)	93 (22.9)	1		1	
GP with master’s or doctoral degree	50 (10.9)	18 (36.0)	1.90 (1.02–3.54)	0.043*	1.83 (0.82–4.08)	0.140
Occupation						
GP and non-university staff (R)	356 (77.9)	82 (23.0)	1		1	
GP and university staff	56 (12.3)	18 (32.1)	1.58 (0.86–2.92)	0.142	0.63 (0.27–1.51)	0.300
Specialist residency	45 (9.8)	11 (24.4)	1.08 (0.53–2.23)	0.833	0.41 (0.16–1.02)	0.056
Department						
Community health centre (R)	162 (35.4)	40 (24.7)	1			
Emergency unit	136 (29.8)	30 (22.1)	0.86 (0.50–1.48)	0.594		
Others	159 (34.8)	41 (25.8)	1.06 (0.64–1.75)	0.821		
Type of workplace						
Community health centre (R)	95 (20.8)	19 (20.0)	1			
Private clinic or hospital	191 (41.8)	48 (25.1)	1.34 (0.74–2.45)	0.336		
Government hospital	171 (37.4)	44 (25.7)	1.39 (0.75–2.55)	0.293		
Location of workplace						
District (R)	137 (30.0)	26 (19.0)	1		1	
Regency	184 (40.3)	46 (25.0)	1.42 (0.83–2.45)	0.202	1.37 (0.78–2.41)	0.275
Province	136 (29.8)	39 (28.7)	1.72 (0.98–3.02)	0.061	1.70 (0.88–3.28)	0.116
Attended province-level conference						
Yes	302 (66.1)	79 (26.2)	1.36 (0.85–2.17)	0.194	1.19 (0.71–1.99)	0.506
No (R)	155 (33.9)	32 (20.6)	1		1	
Attended national conference						
Yes	160 (35.0)	47 (29.4)	1.51 (0.98–2.35)	0.064	1.18 (0.70–1.98)	0.540
No (R)	297 (65.0)	64 (21.5)	1		1	
Attended international conference						
Yes	32 (7.0)	11 (34.4)	1.70 (0.79–3.65)	0.172	1.04 (0.44–2.46)	0.937
No (R)	425 (93.0)	100 (23.5)	1		1	
Medical practice experience (years)						
Less than 5 years (R)	303 (66.3)	64 (21.1)	1		1	
5 years or more	154 (33.7)	47 (30.5)	1.64 (1.06–2.55)	0.028*	1.28 (0.63–2.64)	0.496
Had contact with patient(s) presenting signs and symptoms of Zika virus infection						
Yes	36 (7.9)	18 (50.0)	3.53 (1.76–7.05)	< 0.001**	3.14 (1.49–6.58)	0.003**
No (R)	421 (92.1)	93 (22.1)	1		1	

*aOR* Adjusted odds ratio, *CI* Confidence interval, *OR* Odds ratio, *R* Reference group

* Significance at 0.05, ** Significance at 0.01

### Correlation and association between knowledge and attitude

We found that there was a significant but weak positive correlation between knowledge and attitude towards pregnancy-related issues of ZIKV infection (*r* = 0.09; 95% CI: 0.02–0.18, *P* = 0.037). This indicates that attitude was increasingly positive with greater knowledge. To validate this result, further analysis was conducted using knowledge and attitude scores that had been classified as “good” and “poor” based on a cut-off point of 75%. Our analysis indicated that there was no impact of knowledge on attitude using this classification system (OR: 1.41, 95% CI: 0.88–2.25, *P* = 0.154).

## Discussion

General practitioners are frontline HCWs in Indonesia and other countries, and their ability to comprehensively react to cases of Zika disease in their communities is, in part, limited by the knowledge and attitudes that they hold. Studies on knowledge and attitude towards ZIKV infection among GPs are limited. Given the fact that HCWs have critical roles in the prevention and management of ZIKV infection, this study was conducted to fill this gap in the literature. We found that almost 40% of the participating GPs had a poor knowledge of pregnancy-related issues of ZIKV infection. For example, 73.9% of respondents mentioned that ZIKV could be passed through breastfeeding. In fact, there are no reports to suggest that a ventricular septal defect was associated with ZIKV infection, and the U.S. CDC stated that there are no reports of infants getting ZIKV through breastfeeding, and mothers are encouraged to breastfeed even in areas with risk of Zika [67]. One of the plausible explanations of this finding is that ZIKV infection has not been a subject taught in Indonesia. For instance, Zika was not listed in the 2006 National General Practitioner Competence List of the Republic of Indonesia [68]. Therefore, while a few medical schools in Indonesia have incorporated the subject into their curriculum, this knowledge is not a mandatory subject for medical doctor graduates in Indonesia [69]. In addition, we found no independent factor that was significantly associated with knowledge among GPs. Previously, our study in Aceh province found that specialists had significantly lower knowledge compared to GPs, and some determinants relating to specialists were associated with lower knowledge [51]. For example, doctors whose workplace had access to medical journals (which was the case for most of the specialists) had lower knowledge compared to doctors whose workplace had restricted access to journals (e.g., in primary health service). In addition, doctors who worked in community health centres had greater knowledge compared to doctors who were working in private clinics or private hospitals and government hospitals, where most doctors were specialists. Therefore, it is not surprising that none of the independent variables were associated with knowledge in this survey because all of the respondents were GPs with a homogenous knowledge base. All of the respondents of this study received their medical education from the national curricula based on the 2006 National General Practitioner Competence List [68], where Zika was not listed as a compulsory disease.

We found that 66.3% of the participants had a medical practice experience of less than 5 years, and only 7.9% of the total participants stated that they had been contacted by a patient presenting signs and symptoms of ZIKV infection. These characteristics suggest that most of our participants could be considered to be at an intermediate level of expertise instead of an expert level [70]. Doctors at an intermediate level of expertise are better at recalling more signs and symptoms because they are able to focus on isolated signs and symptoms, and connect them to a pathophysiology mechanism [70]. In contrast, experts commonly use encapsulated knowledge or short-cut knowledge which is constructed from the memories of previous patients, which creates illness scripts. As a consequence, the expert may be inhibited in recalling signs and symptoms [70]. This could explain why most of our participants had relatively good knowledge about ZIKV infection compared to the specialist doctors in our previous survey [51]. Yet, the GPs could also be categorized as having incomprehensive knowledge about ZIKV infection since there were misperceptions about several aspects of the infection (i.e., that ZIKV could be transmitted via breastfeeding and that ventricular septal defect could be caused by the infection).

This incomprehensive knowledge, interestingly, was not adequate to improve GPs’ attitude towards ZIKV infection management because attitude change requires more than merely cognitive or knowledge transformation but also relies on affective and behavioural dimensions of change [71]. Attitude is a summary evaluation of an object of thought that is constructed in a particular situation; it is context-dependent [71]. To improve GPs’ attitude towards ZIKV infection, more exposure to cases of ZIKV infection is therefore required, to provide some experience enabling them to acquire diagnostic and management knowledge and to apply their knowledge in a practical setting [72]. The lack of exposure of real cases of ZIKV infection produces fewer opportunities for the GPs to apply their relatively good knowledge on ZIKV infection, and as a result the knowledge generates less of an impact on their attitudes. This might, in part, explain the lack of an association between knowledge and attitude among GPs.

This study revealed that GPs who had had contact with patient(s) presenting signs and symptoms of ZIKV infection were three times more likely to have a positive attitude. This finding could be explained by the experiential learning theory, in which learners will have better perception and cognition towards a disease if they are directly exposed to actual cases [73].

In general, our present study together with our previous survey [51] indicates that knowledge of ZIKV infection in HCWs in Indonesia is relatively low. Therefore, strategies for enhancing the capacity of HCWs (including GPs and other healthcare staffs) to respond to ZIKV infection may be needed. Scholars in the field of knowledge management have focused on knowledge seeking behavior of GPs at the global level. One study described a preference of GPs for local knowledge, for example, transfers from their own experiences or colleagues’ experiences and communication with leaders in their institution, as a reaction to the overwhelming information overload from 400.000 medical articles published each year [74]. In addition, GPs preferred interactive sessions in workshops and conferences over didactic lectures to improve their performance in clinical decision making [75, 76]. Therefore, efforts to increase GPs’ knowledge on ZIKV infection should be supported by incorporating relevant information regarding ZIKV infection in continuing medical education (CME) programmes in collaboration with the Indonesian Medical Council which regulates future GPs’ competence and certification.

Online survey research has advantages which include time and cost benefits, however, this study is not without limitations. The results from this study, therefore, should be interpreted with caution. First, the incomplete response rate in this survey is high (46.3%) if compared to our previous survey [51, 56]. Second, there is potential for biased geographical selection of respondents because certain localities in Indonesia are less likely to have internet access than others. Third, we did not collect the exact location of the respondents making it not possible to create a map showing spatial heterogeneity. However, our study is still able to show the distribution of the respondents between rural (district) and urban (regency) areas. In addition, we also analyzed respondents based on type of workplace, which is one of the most important heterogeneity analysis in medical setting because it reflects facility characteristics. Finally, dishonesty can be an issue in the sense that some respondents may not have been fully truthful with their answers, or may have looked up the correct response to answers. To minimize this last issue, a clear introduction on the first page of the survey was provided asking participants to respond based on their current knowledge and beliefs without trying to find the correct answer from other resources.

## Conclusions

This study reveals that the knowledge about pregnancy-related problems with ZIKV infection is relatively high and homogenous among GPs across the most populous regions of Indonesia. However, GPs in Indonesia still have a poor attitude towards pregnancy-related problems of ZIKV infection, although contact experience with a patient presenting signs and symptoms of ZIKV infection was associated with a more positive attitude. Strategies for enhancing the capacity of GPs in Indonesia to respond to ZIKV infection are needed. Although the global emergency regarding ZIKV infection has been lifted, Indonesia is still at risk for ZIKV transmission, rendering medical education about ZIKV highly important and relevant for prevention and control efforts. One of the efforts with long-term effect will be to include the prevention and management of ZIKV infection in the National General Practitioner Competence List of Indonesia.

## Additional files

Additional file 1: Completed of STROBE checklist of the study. (PDF 478 kb)

Additional file 2: Detailed questions used to assess knowledge and attitude domain. (PDF 342 kb)

## Data Availability

The datasets used and/or analyzed during the current study are available from the corresponding author on reasonable request.

## Abbreviations

aOR: Adjusted odds ratio
CDC: Centers for Disease Control and Prevention
CME: Continuing medical education
GP: General practitioners
HCW: Healthcare worker
OR: Crude odds ratio
R: Reference category
r_s_: Spearman’s rank correlation
SPSS: Statistical Package of Social Sciences
ZIKV: Zika virus
