# Supplementary material for: Knowledge and attitude towards pregnancy-related issues of Zika virus infection among general practitioners in Indonesia
Source: BMC Infect Dis. 2019 Aug 6;19:693. doi: 10.1186/s12879-019-4297-4 (PMC6683397; doi:10.1186/s12879-019-4297-4)
Supplement: Supplementary file 1 — Completed of STROBE checklist of the study. (PDF 478 kb) [file 12879_2019_4297_MOESM1_ESM.pdf]

Additional file 1. Completed of STROBE checklist of the study

STROBE Statement - Checklist of items that should be included in reports of cross-sectional studies

Items	Item No	Recommendation	Subheading of manuscript
**Title and abstract**	1	(a) Indicate the study's design with a commonly used term in the title or the abstract<br>*Community acceptance and willingness-to-pay for a hypothetical Zika vaccine: A cross-sectional study in Indonesia*	*Title*
		(b) Provide in the abstract an informative and balanced summary of what was done and what was found<br>*Abstract in this study consisting of background, method, results and conclusion sections with informative and balanced information.*	*Summary*
**Introduction**			
Background/rationale	2	Explain the scientific background and rationale for the investigation being reported<br>*We provided specific background and reasons for conducting this study. One of the main reasons is although studies have been conducted to assess the acceptance of Zika vaccine, no findings related to WTP for a Zika vaccine are available currently.*	*Introduction*
Objectives	3	State specific objectives, including any prespecified hypotheses (N/A)<br>*The aim of this study assess the community acceptance and WTP for a hypothetical ZV and associated modifiable determinants in Aceh and West Sumatra provinces of Indonesia.*	*Introduction*
**Methods**			
Study design	4	Present key elements of study design early in the paper<br>*"A health facility-based cross-sectional study was conducted in Aceh and West Sumatra province from 1 February to 13 June 2018"*	*Methods: Study design, setting and location*
Setting	5	Describe the setting, locations, and relevant dates, including periods of recruitment (N/A), exposure (N/A), follow-up (N/A), and data collection.<br>***Setting of study:*** *Health facility-based cross-sectional study.* ***Locations of study:*** *Study was conducted in Aceh and West Sumatra province. Both provinces are located in Sumatra Island which is situated in the westernmost region of the Indonesian archipelago. The study was conducted in seven regencies or municipalities of Aceh (Banda Aceh, Bireun, Aceh Utara, Aceh Selatan, Lhokseumawe, Aceh Jaya, Aceh Besar) and three regencies of West Sumatra (Padang Panjang, Tanah Datar and Solok).* ***Relevant dates of study or data collection:*** *1 February to 13 June 2018.* ***Data collection:*** *Structured interviews assisted by a validated questionnaire were conducted in Bahasa Indonesia to collect the information of interest from respondents.*	*Methods: Study design, setting and location*<br><br>*Methods: Interview and data collection*
Participants	6	Give the eligibility criteria, and the sources and methods of selection of participants<br><br>***Eligible criteria:*** *Participants who were married, have had children or were expecting their first child during the study, had resided in the specified regency or municipality for more than 3 months, and were able to communicate in Bahasa Indonesia (the national language) were considered to be eligible for inclusion.* ***Method used to select the participant:*** *Eleven out of 42 regencies or municipalities in both provinces were purposefully selected to include both urban and suburban areas and participants were approached and recruited via a convenience sampling method.*	*Methods: Sampling and sample size*

Items	Item No	Recommendation	Subheading of manuscript
Variables	7	Clearly define all outcomes, exposures (N/A), predictors, potential confounders, and effect modifiers (N/A). Give diagnostic criteria (N/A)<br>***Response variables:*** *Acceptance and willingness to pay for a Zika vaccine*<br>***Explanatory variables****: Sociodemographic data such as age, sex, educational attainment, employment status, types of workplace, monthly income, numbers of children, had heard about Zika infection prior interview and attitude for childhood vaccination*	*Methods: Study variable*
Data sources/ measurement	8*	For each variable of interest, give sources of data and details of methods of assessment (measurement). Describe comparability of assessment methods if there is more than one group.<br>*All interest variables (included dependent and independent variables) I this study were obtained from face-to-face interview.*	*Methods: Interview and data collection*
Bias	9	Describe any efforts to address potential sources of bias<br>*The possible bias of this study was from type of data that was obtained. Therefore a series of diagnostic assessments was conducted to check how well the data met the model assumptions used in the multivariate model using Variance Inflation Factor (VIF), Lagrange multiplier test, Glejser test and Kolmogorov-Smirnov test to assess multicollinearity, autocorrelation, heteroscedasticity and residual normality, respectively.*<br><br>*In addition, confounding factors were explored between the adjusted odds ratio (aOR) in multivariate analyses and the crude odds ratio (OR) in univariate analyses using strategy that have been described previously.*	*Methods: Statistical analysis*
Study size	10	Explain how the study size was arrived at<br>*"Based on the population of both provinces in 2017 (10·51 million) the minimum sample size required was 385, based on the assumption that the acceptability rate was conservatively estimated to be 50% with a 5% margin of error and a confidence interval of 95%."*	*Methods: Sampling and sample size*
Quantitative variables	11	Explain how quantitative variables were handled in the analyses. If applicable, describe which groupings were chosen and why.<br>*"For statistical analysis, the total raw score for the attitude towards childhood vaccination was converted to a 0–100 scale and the then dichotomized into two categories: good attitude (score <50) and poor attitude (score ≥50), following previous literature [37-40]. The score of acceptance to ZV were computed as the sum of the response scores from two questions giving the additive scale scores ranged from 0 to 4. The acceptance was then categorized into "willing" and "not willing" based on a 75% cut-off point (i.e. score 3 or more classified as "willing")."*	*Methods: Statistical analysis*
Statistical methods	12	(*a*) Describe all statistical methods, including those used to control for confounding<br>*"To assess the explanatory variables influencing participants' ZV acceptance, a logistic regression was employed. The estimated odds ratio (OR) was interpreted in relation to one of the categories, which was designated as the reference category (R).""Explanatory variables influencing participants' WTP were determined using a multivariate linear regression model"*<br>*Detailed can be found in Statistical analysis*	*Methods: Statistical analysis*
		(*b*) Describe any methods used to examine subgroups and interactions<br>*We divided attitude towards childhood vaccination into the individual sub-domain.*	*Methods: Statistical analysis*<br><br>*Results: Table 1 and Table 2*
		(*c*) Explain how missing data were addressed<br>*In this study, we only included data of participants who provided or completed all section of the questionnaire. All participants with missing data were excluded from analyses.*	*Result: Study population characteristics*
		(*d*) If applicable, describe analytical methods taking account of sampling strategy<br>*Not applicable but the analytical analysis in this study was choose based on distribution of our data.*	*Methods: Statistical analysis*
		(*e*) Describe any sensitivity analyses (N/A)	

Items	Item No	Recommendation	Subheading of manuscript
**Results**			
Participants	13*	(a) Report numbers of individuals at each stage of study—eg numbers potentially eligible, examined for eligibility, confirmed eligible, included in the study, completing follow-up (N/A), and analysed.<br>*"We approached and interviewed 1,102 respondents in eleven regencies and 145 (13·2%) refused to participate or had incomplete interviews. Most participants discontinued the interview when they were called by a nurse to meet a doctor. A total of 956 (86·8%) participants were analyzed for ZV acceptance."*	*Results: Study population characteristics*
		(b) Give reasons for non-participation at each stage<br>*In this study, the non-participant occurred in one stage only which was incomplete data during data collection. All incomplete data from participants were excluded from the analysis.*	*Results: Study population characteristics*
		(c) Consider use of a flow diagram	
Descriptive data	14*	(a) Give characteristics of study participants (eg demographic, clinical, social) and information on exposures and potential confounders<br>*In this study, the characteristics of the participants are depicted in Table 1. We included a very little information of the Table 1 into description text to avoid repetitive.*	*Results: Study population characteristics*
		(b) Indicate number of participants with missing data for each variable of interest<br>*In this study, we only included data of participants who provided or completed all section of the questionnaire. Meaning that each variable of interest had the same number of participants.*	*Results: Study population characteristics*
Outcome data	15*	Report numbers of outcome events or summary measures<br>*In this study, the acceptance was then categorized into "willing" and "not willing" based on a 75% cut-off point (i.e. score 3 or more classified as "willing") and the mean and median of WTP were estimated. The number of each categories and the WTP were presented in the Result section.*	*Methods: Statistical analysis*<br><br>*Results: Acceptance of Zika vaccine and associated explanatory variables & Willingness-to-pay for a Zika vaccine*
Main results	16	(*a*) Give unadjusted estimates and, if applicable, confounder-adjusted estimates and their precision (eg, 95% confidence interval). Make clear which confounders were adjusted for and why they were included.<br>*In this study, unadjusted estimates (univariate analysis) and adjusted estimates are calculated for each explanatory and response variable and both of them provided in Table 1.*	*Table 1*
		(*b*) Report category boundaries when continuous variables were categorized<br>*"The acceptance was then categorized into "willing" and "not willing" based on a 75% cut-off point (i.e. score 3 or more classified as "willing"). The results were given in Table 1*	*Methods: Statistical analysis*<br><br>*Result: Table 1*
		(*c*) If relevant, consider translating estimates of relative risk into absolute risk for a meaningful time period	
Other analyses	17	Report other analyses done—eg analyses of subgroups and interactions, and sensitivity analyses (N/A)	*N/A*
**Discussion**			
Key results	18	Summarise key results with reference to study objectives<br>*The key findings are explained throughout the discussion section with comparison with other studies.*	*Discussion*
Limitations	19	Discuss limitations of the study, taking into account sources of potential bias or imprecision. Discuss both direction and magnitude of any potential bias.<br>*Here we discussed the limitations of our study.*	*Discussion*
Interpretation	20	Give a cautious overall interpretation of results considering objectives, limitations, multiplicity of analyses, results from similar studies, and other relevant evidence.<br>*Some cautious are given in the discussion related to our finding related to our study limitations.*	*Discussion*

Items	Item No	Recommendation	Subheading of manuscript
Generalisability	21	Discuss the generalisability (external validity) of the study results.<br>*Some generalisabilities of the results from this study were discussed especially in the larger context of Indonesia.*	*Discussion*
**Other information**			
Funding	22	Give the source of funding and the role of the funders for the present study and, if applicable, for the original study on which the present article is based.<br><br>*There was no funding related to this study. However, some authors supported with some funding.*	*Acknowledgement*

Note: An Explanation and Elaboration article discusses each checklist item and gives methodological background and published examples of transparent reporting. The STROBE checklist is best used in conjunction with this article (freely available on the Web sites of PLoS Medicine at http://www.plosmedicine.org/, Annals of Internal Medicine at http://www.annals.org/, and Epidemiology at http://www.epidem.com/). Information on the STROBE Initiative is available at www.strobe-statement.org.
