# Supplementary material for: Knowledge and attitude towards pregnancy-related issues of Zika virus infection among general practitioners in Indonesia
Source: BMC Infect Dis. 2019 Aug 6;19:693. doi: 10.1186/s12879-019-4297-4 (PMC6683397; doi:10.1186/s12879-019-4297-4)
Supplement: Supplementary file 2 — Detailed questions used to assess knowledge and attitude domain. (PDF 342 kb) [file 12879_2019_4297_MOESM2_ESM.pdf]

Additional file 2. Detailed questions used to assess knowledge and attitude domain

Knowledge on Zika infection

No	Question
1	A pregnant woman already infected with Zika virus can pass Zika virus to her foetus during pregnancy through placenta
2	A mother can pass Zika virus to her child through breastfeeding
3	Zika infection during pregnancy can cause foetuses to have a birth defect
4	Zika infection during pregnancy can cause foetuses to have a birth defect of the hearth called Ventricular Septal Defect (VSD)
5	Zika infection during pregnancy can cause foetuses to have a birth defect of the brain called microcephaly

Attitude towards Zika infection

No	Question
1	Do you think a pregnant woman with laboratory evidence of Zika virus infection should undergo caesarean section to prevent intra uterine infection?
2	Do you think infants born from mothers with possible Zika virus infection during pregnancy should receive a comprehensive physical examination, serological and molecular testing of the Zika virus?
3	Do you think a pregnant woman with acute Zika virus infection should be treated with antibiotics
4	Do you think a pregnant woman with acute Zika virus infection should be treated with antiviral drugs?
